# Autoimmune Pancreatitis Type 2: Case Report

**DOI:** 10.1177/2324709617734245

**Published:** 2017-10-10

**Authors:** Chidinma Onweni, Harika Balagoni, Jennifer M. Treece, Emmanuel Addo Yobo, Archi Patel, Jennifer Phemister, Manoj Srinath, Mark F. Young

**Affiliations:** 1 East Tennessee State University, Johnson City, TN, USA; 2 James H. Quillen Veterans Affairs Medical Center, Mountain Home, TN, USA

**Keywords:** autoimmune pancreatitis, type 1, type 2, corticosteroid therapy, IgG4 antibody, IgG4-related disease, chronic pancreatitis, lymphoplasmacytic sclerosing pancreatitis

## Abstract

A middle-aged man presents with acute pancreatitis of unknown etiology and is found to have a presentation consistent with the diagnosis of type 2 autoimmune pancreatitis (AIP). AIP is a group of rare heterogeneous diseases that are challenging to diagnose. There are 2 types of AIP. Type 1 disease is the more common worldwide than type 2 AIP. While type 1 AIP is associated with IgG4-positive antibodies, type 2 AIP is IgG4 antibody negative. Both types of AIP are responsive to corticosteroid treatment. Although type 1 AIP has more extrapancreatic manifestations and more commonly relapses, this is a case of a patient with type 2 AIP with inflammatory bowel disease and relapsing course.

## Case Report

**Figure 1.**
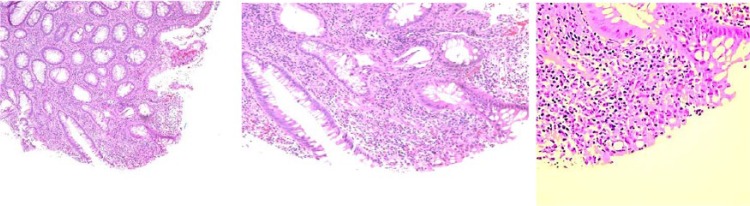
Rectosigmoid colon, biopsy: Acute and chronic inflammation, cryptitis, crypt abscesses, crypt distortion, and crypt drop-out. Focal surface ulceration is also noted on the colon biopsy, showing infiltrates consistent with colitis. No dysplasia identified.

We report a case of a 64-year-old male patient who presented with acute pancreatitis. The patient’s history of present illness, past medical history, recent social history, right upper quadrant ultrasonography, and hepatitis panel were all nonsuggestive of an etiology for the patient’s acute pancreatitis. Of note, the patient admitted to a remote smoking history, remote heavy drinking as a teenager, and denied illicit drug use. Magnetic resonance cholangiopancreatography showed dilated intrahepatic and extrahepatic bile ducts and a distal common bile duct (CBD) stricture with a moderately enlarged head of the pancreas. Laboratory studies collected during the hospital admission were negative for anti-mitochondrial antibody, negative for anti-smooth muscle antibody, negative for immunoglobulin G (IgG) subclasses, antinuclear antibody (ANA) was negative, and the hepatitis panel was also negative. The patient’s symptoms improved gradually, so the patient was discharged home without a definitive diagnosis for the underlying etiology of his acute pancreatitis. A few days following hospital discharge home, the patient reported new onset of persistent diarrhea. Initial fixed sigmoidoscopy showed circumferential lesion suggesting a diagnosis of ulcerative colitis. A subsequent colonoscopy showed skipped lesions suggesting a diagnosis of Crohn’s disease. The ileum was not involved but rectum was involved. Because the patient’s colon lesion and histological evaluation suggested both ulcerative colitis and Crohn’s disease, the diagnosis of indeterminate colitis was determined as neither was completely met (see Figure 1).

A few weeks later, he underwent an endoscopic ultrasound (EUS), which showed a pancreatic head mass. Core biopsy of the pancreatic mass revealed benign glandular cells, rare mildly atypical ductal cells, few plasma cells, few inflammatory cells, and stains for immunoglobulin G4 (IgG4) and CD138 demonstrated nonspecific staining, which were inconclusive. CD138 is known to be a plasma cell marker, but pancreatic acinar cells in chronic pancreatitis also express CD138.^1^ Background debris with no malignant cells was also identified. The patient was treated with intravenous (IV) corticosteroids with a slow taper of oral corticosteroids over several months. After completing the corticosteroid course, a repeated EUS was done approximately 6 months following the first EUS, which was normal with no evidence of a dilated CBD; the pancreatic head parenchyma appeared normal and there was no evidence of a focal mass lesion or progression. This implies that the patient’s pancreatitis was responsive to corticosteroids, which indicates a diagnosis of an autoimmune cause.^2^ Type 2 AIP was diagnosed as this patient’s pancreatitis was IgG4 negative,^2^ associated with inflammatory bowel disease,^3^ and is associated with mass in the head of the pancreas,^4^ which was also present in this patient before treatment with corticosteroids.

Since his initial diagnosis of type 2 AIP, the patient has had multiple recurrences of diarrhea and some recurrence pancreatitis following cessation of corticosteroid use. He was evaluated by multiple experts at several facilities, who recommended focusing treatment on the patient’s indeterminate colitis with immunomodulators. The patient was initially started on adalimumab (Humira) with improvement of colitis and control of recurrent (one episode of recurrent) acute pancreatitis, which allowed for tapering the patient off the chronic corticosteroids. His diarrhea later returned, and he was found to have elevated antibodies to adalimumab, so adalimumab was discontinued and vedolizumab has been started, which is currently controlling the patient’s symptoms.

## Epidemiology

Autoimmune pancreatitis is a group of rare heterogeneous diseases. There are 2 types of AIP. Type 1 disease is the more common worldwide than type 2 AIP.^4^ Type 1 AIP is more common in men and occurs in patients that are approximately 60 years older, and type 2 AIP presents in patients that are 40 to 50 years old and occurs at the same frequency in males and females.^4^

## Pathology

Type 1 AIP is associated with elevated levels of IgG4-positive cells and extrapancreatic manifestations.^5^ The histology of both type 1 and type 2 AIP includes periductal lymphoplasmacytic infiltrate and storiform fibrosis, but type 2 also has granulocytic epithelial lesion duct change. The pancreatic histology of type 1 AIP is known as lymphoplasmacytic sclerosing pancreatitis with obliterative phlebitis but without predominant neutrophilic infiltrate in the lobule and duct.^2^ In type 2 AIP, IgG4-positive cells are uncommon, IgG is normal, predominant neutrophilic infiltrate in the lobule and duct are present, and it is more difficult to diagnose than Type 1 AIP^7^ (see Table 1).

**Table 1.** Autoimmune Pancreatitis, Type 1 and Type 2^3-8^.

	Type 1	Type 2
Prevalence	More common^4^	Less common^4^
Signs/symptoms	Obstructive jaundice, weight loss^7^	Obstructive jaundice, abdominal pain, weight loss, acute pancreatitis^6^
Patient demographics	More common in men,^4^ mean age of presentation is in sixth decade of life^8^	Younger patients—present around 40-50 years old,^4^ equal between men and women
Serum IgG	High^7^	Normal^7^
IgG4 autoantibody	Positive^4^	Negative^4^
IgG4-positive plasma cells	Many^4^	None or very few (<10 cells/high-power field)^4^
Extrapancreatic manifestations	More common (50%)^4^	Less common^4^
	Sclerosing cholangitis^7^	Associated with inflammatory bowel disease^4^
Imaging	Enlargement of the pancreas, diffuse or focal; narrowing of the bile and pancreatic ducts^4^	Mass of pancreatic head^4^
Pancreatic histology	Lymphoplasmacytic sclerosing pancreatitis, periductal lymphoplasmacytic infiltrate, storiform fibrosis (swirling pattern), obliterative phlebitis, and IgG4-positive plasma cells^5^	Granulocytic epithelial lesion duct change; periductal lymphoplasmacytic infiltrate and storiform fibrosis; predominant neutrophilic infiltrate in the lobule and duct are present^3^
Differential diagnosis	Pancreatic ductal adenocarcinoma	Pancreatic ductal adenocarcinoma
Diagnosis	Imaging and serological markers^4^	Core needle biopsy for histological confirmation^4^
Treatment	Corticosteroids and immunomodulators (eg, rituximab)^6^	Corticosteroids and immunomodulators (eg, rituximab)^6^
Relapses	More common (35% to 60%)—needs immunomodulators to maintain remission^6^	Less common (<10%)^6^
Prognosis	Good^6^	Good^6^

The HISORt diagnostic criteria for diagnosing type 1 AIP is shown in Table 2. HISORt stands for histology, pancreatic imaging, serology, other organ involvement, and response to therapy.^9^

**Table 2.** The Mayo Clinic HISORt Criteria to Diagnose Type 1 AIP^9^.

Diagnostic Feature	Criteria
*H*istology	At least one of the following histological findings:
	● Periductal lymphoplasmacytic infiltrate with obliterative phlebitis and storiform fibrosis
	● Lymphoplasmacytic infiltrate with storiform fibrosis showing abundant (≥10 cells/high power field) and IgG4-positive cells
Pancreatic *I*maging	● *Typical image*:
	● Diffusely enlarged gland with delayed enhancement; diffusely irregular, attenuated main pancreatic duct
	● *Others*:
	● Focal pancreatic mass/enlargement; focal pancreatic duct stricture; pancreatic calcification; pancreatitis; or pancreatic atrophy
**S**erology	Elevated serum IgG4 level
Other *Or*gan involvement	Hilar/intrahepatic biliary strictures, persistent distal biliary stricture, parotid/lacrimal gland involvement, mediastinal lymphadenopathy, retroperitoneal fibrosis
*R*esponse to steroid *t*herapy	Resolution or marked improvement of pancreatic/extrapancreatic manifestations with steroid therapy

## Clinical Presentation and Diagnosis

Both types of AIP can present with obstructive jaundice and weight loss. Type 2 AIP more commonly presents with obstructive jaundice, abdominal pain, weight loss, and acute pancreatitis.^6^ Patients with AIP present with chronic pancreatitis of unknown etiology. Extensive evaluation for underlying etiology of pancreatitis is indeterminate. AIP is seldom encountered and is not a definitive diagnosis. Therefore, some investigators have suggested using a combination of laboratory testing, imaging, histological findings, association to other autoimmune disease, and response to corticosteroid treatment to establish the diagnosis.^10^ Type 1 AIP can be diagnosed through imaging and serological markers positive for IgG4 autoantibodies. Type 2 AIP requires core needle biopsy to diagnose through localizing the granulocytic epithelial lesion of the pancreatic duct.^4^

## Treatment

AIP is responsive to corticosteroid use in the acute setting, but relapse is common following cessation of corticosteroid use. Recurrence is more common in type 1 AIP but can occur in type 2 AIP. Immunomodulators are effective in maintaining remission.^6^ For the patient presented in this case, the patient took repeated courses of corticosteroid with recurrence of symptoms on cessation of corticosteroid treatment. An expert at a prestigious university hospital recommended that the patient’s treatment be directed toward colitis with biologic therapy as it was thought that biologic may also provide benefit to AIP. The patient was started on Humira and was tapered off prednisone. Six months later, the patient was experiencing abdominal pain and diarrhea, stool studies were positive for *Clostridium difficile*. At that time Humira drug levels and antibody levels were drawn, which showed elevated antibody levels. The patient completed a course of metronidazole with resolution of symptoms. The patient was previously on azathioprine but developed hepatotoxicity with liver function tests and returned to normal after medication discontinuation.

## Discussion

The purpose of this case report is to review the limited knowledge that exists about AIP, particularly type 2 AIP, which is rarer than type 1 AIP, and to discuss how AIP presents clinically with methods to diagnosis this elusive disease. Type 1 AIP is the variant of AIP that has been described and studied more extensively as it is more common than type 2 AIP.^2^ The current diagnostic criteria focus on diagnosing type 1 AIP without assessing for type 2 AIP. From this case, we learned that there is limited knowledge about the diagnosis of type 2 AIP. The diagnosis is unclear even following biopsy of the pancreas. As seen in this patient, the biopsy of the pancreatic mass only revealed benign glandular cells and background debris with no malignant cells identified. The diagnosis was presumed to be type 2 AIP due to IgG4 being negative, the patient responded to corticosteroid treatment with improvement of symptoms, and the resolution of the dilated intrahepatic and extrahepatic bile ducts, the distal CBD stricture, and the mass at the head of the pancreas following corticosteroid use. Of note, the sensitivity of serum IgG4 ranges from 53% to 95%, so having a negative IgG4 level does not exclude type 1 AIP.^11^ It is interesting to note that type 1 AIP is associated with biliary findings, primarily primary sclerosing cholangitis. Type 2 AIP principally affects the pancreas, with only one third having extrapancreatic magnifications, such as inflammatory bowel disease like ulcerative colitis. Our patient, interestingly, fits into the one third of type 2 AIP cases with extrapancreatic findings as he developed persistent diarrhea. He was diagnosed with colitis, and the type of colitis was unclear via biopsy. The gastroenterologist diagnosed him as presumptive ulcerative colitis.

## Conclusion

AIP is a rare disease with type 2 AIP being rarer than type 1 AIP. Diagnosing AIP is challenging as even a core needle biopsy of a pancreatic mass is not useful in diagnosing AIP.^2^ AIP remains a rare disease and little is known about the diagnosis or underlying etiology, especially in type 2 AIP. Type 1 AIP has IgG4-positive antibodies. As relapse is very common following cessation of corticosteroid use treatment, immunomodulators are needed to maintain remission for both type 1 and type 2 AIP.
